# Exploring post-SEPSIS and post-COVID-19 syndromes: crossovers from pathophysiology to therapeutic approach

**DOI:** 10.3389/fmed.2023.1280951

**Published:** 2024-01-05

**Authors:** Darcy Holmes, Marta Colaneri, Emanuele Palomba, Andrea Gori

**Affiliations:** ^1^Infectious Diseases Unit, Foundation IRCCS Ca’ Granda Ospedale Maggiore Policlinico, Milan, Italy; ^2^Department of Infectious Diseases, Luigi Sacco Hospital, Milan, Italy; ^3^Centre for Multidisciplinary Research in Health Science (MACH), University of Milan, Milan, Italy

**Keywords:** post-sepsis syndrome (PSS), post-acute sequelae of COVID-19 (PASC), immune dysfunction, renin-angiotensin system (RAS), angiotensin-converting enzyme 2 (ACE2)

## Abstract

Sepsis, driven by several infections, including COVID-19, can lead to post-sepsis syndrome (PSS) and post-acute sequelae of COVID-19 (PASC). Both these conditions share clinical and pathophysiological similarities, as survivors face persistent multi-organ dysfunctions, including respiratory, cardiovascular, renal, and neurological issues. Moreover, dysregulated immune responses, immunosuppression, and hyperinflammation contribute to these conditions. The lack of clear definitions and diagnostic criteria hampers comprehensive treatment strategies, and a unified therapeutic approach is significantly needed. One potential target might be the renin-angiotensin system (RAS), which plays a significant role in immune modulation. In fact, RAS imbalance can exacerbate these responses. Potential interventions involving RAS include ACE inhibitors, ACE receptor blockers, and recombinant human ACE2 (rhACE2). To address the complexities of PSS and PASC, a multifaceted approach is required, considering shared immunological mechanisms and the role of RAS. Standardization, research funding, and clinical trials are essential for advancing treatment strategies for these conditions.

## 1 Introduction

Sepsis is defined as a life-threatening organ dysfunction resulting from a dysregulation of the host response to infection (Sepsis-3) (1). Besides bacterial, physiological expressions of viral-induced sepsis might occur, caused by various viruses, including SARS-CoV-2 (2). Bearing in mind that COVID-19 is known to trigger sepsis, both COVID-19 and sepsis lead to immune dysfunction and prolonged illnesses. These sequelae are termed post-sepsis syndrome (PSS), and post-acute sequelae of COVID-19 (PASC). As with many chronic inflammatory diseases, PSS and PASC share striking similarities in clinical and pathophysiological mechanisms (3), with reports suggesting that PASC resembles systemic aspects of bacterial and viral mechanisms of PSS (4). For this reason, there is reasoning for whether they can be considered as the same condition.

Although there are gaps in knowledge regarding the precise mechanisms underpinning these debilitating conditions, here we aim firstly to depict the commonalities between PSS and PASC and secondly to explore potential therapeutic approaches, which are currently lacking both for PSS and PASC. Beyond a mere description of the already reported options, we aim to evaluate the renin-angiotensin system (RAS) as potential therapeutic target. In fact, RAS plays a pivotal role in both PSS and PASC, perpetuating organ imbalance, and modulating immunological and inflammatory mechanisms.

### 1.1 Renin-angiotensin system

RAS is the primary regulator of homeostasis in several organs through intricate interactions with angiotensin-converting enzyme 2 (ACE2) and regulatory mediators. Comprising two opposing arms, a harmful arm involves ACE/Ang II/angiotensin II receptor type 1 (AT1R) pathway, and the protective arm uses ACE2/Ang (1–7)-MasR receptor (MasR). ACE2 is ubiquitously expressed by the endothelium, prominently in the cardiomyocytes, renal tubular cells, intestines, and alveolar epithelial cells. Responsible for converting angiotensin (Ang) I to Ang (1–9) and hydrolysing Ang II to Ang (1–7) (5). Ang II promotes inflammation through increased expression of chemoattractants, enhancing vascular permeability, and inducing vasoconstriction via the breakdown of inflammatory mediators involved in vasodilation. Whereas, Ang (1–7) inhibits these signaling pathways, suppressing the inflammatory response (6).

## 2 Epidemiological and clinical characteristics of PSS and PASC

While no specific definitions exist for PSS or PASC, their remarkable similarities suggest they could be one of the same. Symptoms manifest after the acute phases of sepsis and COVID-19 and persist months after their resolution. The lack of standardized criteria hinders establishment of precise diagnostic criteria, making it difficult to capture the full epidemiologically extent. As survival rates of sepsis and COVID-19 increase, reinfections and readmissions for PSS and PASC rise (7, 9). Regarding sepsis, approximately 50% of patients recover, while around 30% are readmitted within 90 days. Similarly, following COVID-19 recovery, approximately 10–20% of patients are readmitted within 30 to 60 days (9), with PASC afflicts 50% of survivors (10). Efforts to establish a uniform diagnostic criterion and conduct comprehensive studies are essential for understanding PSS and PASC and enabling personalized strategies for prevention and management.

PSS and PASC predominantly feature multi-organ dysfunctions, primarily persistent pulmonary, cardiovascular, renal, and cognitive complications. These follow severe infection, resulting from a network of complex interactions, leading to the defining aspects of PSS (7) and PASC (8); hyperinflammatory responses and immune dysfunction weaken the immune system, irrespective of the viral trigger, contributing to tissue-specific manifestations and systemic organ failure (9). Specifically, fatigue, chest pain, muscle, and joint pain (10, 11) reduced or complete loss of taste and smell affect only PASC (12).

Precise pathophysiological mechanisms remain to be fully elucidated for both conditions and likely vary based on clinical phenotype, insult severity, and specific tissues involved (8).

## 3 Pathophysiological mechanisms in PSS and PASC

Innate immunity starts from the recognition of pathogens through pathogen-associated molecular patterns (PAMPs) and/or damage-associated molecular patterns (DAMPs) by toll-like receptors (TLR), instigating pro-inflammatory signalling through activation of immune cells like monocytes, endothelial cells, natural killer cells, dendritic cells, lymphocytes, and macrophages. These secrete cytokines and chemokines to attract T-cells and neutrophils to the infection site and activate specific immune pathways. As infection progresses, adaptive immune cells, including B plasma cells, produce antibodies and memory cells (13). However, if the innate immune cells fail to clear the pathogen, overactivation of immune responses will trigger hyperinflammatory conditions (8). This uncoordinated and dysfunctional immune response impacts organ homeostasis, and results in long-term sequelae seen in PSS and PASC (6, 8).

Immunosuppression involves dysfunction of antigen-presenting cells, such as macrophages, dendritic cells, T-cells, and B-cells, leading to reduced production of inflammatory cytokines, encompassing immune cell exhaustion, apoptosis, autophagy, endotoxin tolerance, and metabolic changes evidenced by increased Treg activity, monocyte deactivation with reduced HLA-DR expression, and decreased production of inflammatory cytokines (14). Immune dysregulation significantly contributes to systemic and tissue-specific manifestations. In PASC specifically, the ACE2 receptor is crucial in dysregulation of inflammation, immune cell homeostasis, and coagulation pathways (15). Although research on the specific role of monocytes and macrophages in PSS and PASC is limited, they are implicated in hyperinflammation and coagulation abnormalities leading to tissue damage and homeostasis disruption. Studies have shown elevated CD14+ and CD16+ intermediate monocytes and activated myeloid cells in PASC patients’ months after infection, possibly due to hematopoietic progenitor cell programming or T-cell interactions (14).

Viral persistence, tissue damage, chronic antigen exposure, various mechanisms like activation-induced cell death, metabolic exhaustion, epigenetic changes, and the influence of anti-inflammatory cytokines like IL-10 and transforming growth factor-beta (TGF-β) collectively contribute to the immunosuppressive environment and immune cell exhaustion (16). Immune cell exhaustion is pivotal in the pathophysiology of both conditions, where immune cells, particularly T-cells, as well as cytokines and chemokines, exhibit diminished capacity to eliminate infected cells and coordinate immune responses. Markers of T-cell exhaustion include reduced CD8+ cytotoxic T-cells, impaired proliferation of CD4+ T-cells due to Tregs, and reduced cytokine production, leading to a shift toward a Th2-type response and away from Th1-type (17). Th1-cells promote pro-inflammatory cytokines (IFN-γ and IL-12), while Th2-cells push anti-inflammatory, IL-4, IL-5, IL-10, and IL-13. Tregs supress excessive immune responses with inhibitory cytokines while Th17 cells recruit and activate neutrophils, affecting the duration and intensity of inflammation (18).

The initial inflammatory response in sepsis or COVID-19 triggers extensive immune activation. Hyperinflammation occurs when these mechanisms fail to resolve tissue injury, leading to systemic activation characterized by excessive release of cytokines such as IL-1, TNF-α, and IL-17 stimulating immune pathways that can affect cardiovascular, cerebrovascular, and pulmonary tissues (19). Dysregulated inflammatory cytokines, particularly IL-6, IL-1β, IFN-γ, and TNF-α, along with decreased CD4+ and CD8+ T cells, are found in post-acute sequelae of these conditions (15). While PASC shows some similarity to sepsis in cytokine expression, it exhibits distinct patterns with unique risk factors. Dysregulation of the TLR signalling pathway leads to excessive pro-inflammatory cytokine production, which in turn dampens T-cell activity. Metabolic alterations reduce T-cell function and hinders formation of memory cells and optimal B-cell activation. Additionally, tissue-specific macrophages contribute to immune disruption and tissue homeostasis (20). Immune dysregulation is behind the most common and most devastating clinical manifestations of PSS and PASC.

### 3.1 Respiratory manifestations of PSS and PASC

Pulmonary manifestations commonly occur in both PSS (21) and PASC (22) leading to recurrent infections, breathlessness, chronic cough, and long-term lung damage. One of the most prevalent shared symptoms is acute respiratory distress syndrome (ARDS) (13), characterized by severe lung inflammation, increased vascular permeability, pulmonary edema, and acute hypoxemia. Survivors often face long-term complications, such as pulmonary fibrosis, breathing difficulties, and a heightened risk of infections. Pathophysiology of ARDS mainly involves excessive neutrophils migration, systemic hyperinflammatory responses, endothelial dysfunction, and apoptosis. This results in the loss of alveolar-capillary membrane integrity, an overactive innate immune response, abnormal coagulation, impaired gas exchange, and ultimately acute respiratory failure and death (21, 23, 24). Key immune cells, including alveolar epithelial cells, alveolar macrophages, and neutrophils, are vital in producing proinflammatory molecules, therefore regulating them is a potential target for therapeutic strategies in PSS and PASC.

Reduced ACE2 activity in response to infection leads to increased levels of DABK, resulting in the release of pro-inflammatory mediators such as CXCL5, MIP2, KC, TNF-α, and increased neutrophil infiltration. This cascade leads to elevated levels of IL-6, IL-8, TNF-α, and IFN-γ, and is linked to systemic inflammation, longer ICU stays, and a high 90-day mortality. Additionally, systemic immunosuppression is observed in ARDS patients concurrently with persistent pulmonary inflammation (25).

The development and resolution of ARDS are linked to Toll-like receptor (TLR) signaling pathways, which interact with the RAS. Ang II upregulates TLR4 by binding to AT1R, activating proinflammatory pathways and increasing the risk of pneumonia. Conversely, stimulation of the Ang 1-7/ACE2 axis appears beneficial in acute lung injury. ACE2 activity exerts protective effects by regulating inflammatory mediators like IL-1, IL-6, and TNF-𝛼, and improving oxygen levels and pulmonary function. Disruption of RAS equilibrium renders ACE/Ang II/AT1R signaling harmful (26).

### 3.2 Cardiovascular manifestations of PSS and PASC

**Figure 1:**
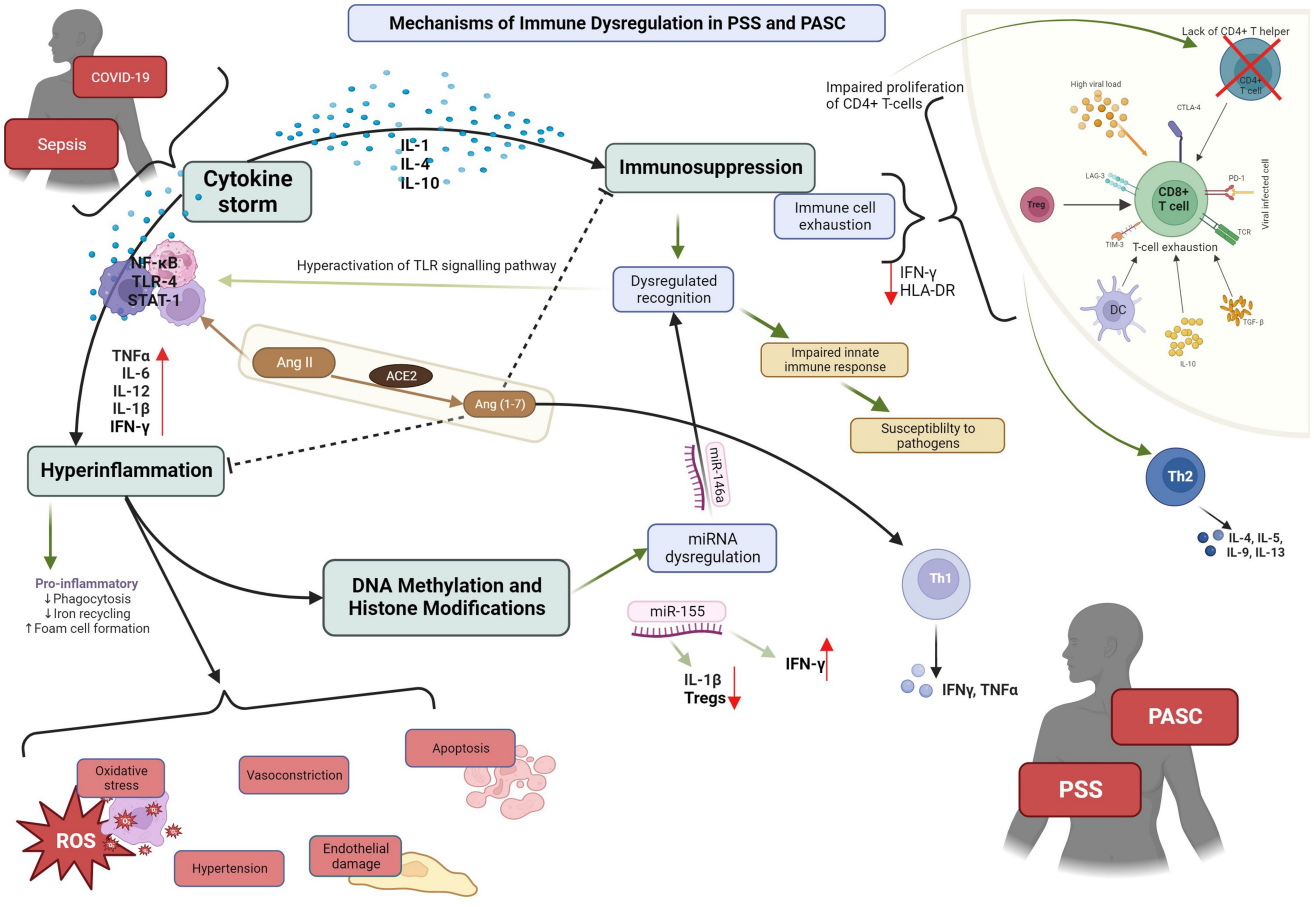
PSS: post sepsis syndrome; PASC: post-acute sequelae of COVID-19. Depiction of the pathophysiological mechanisms and cytokines cascades involved in PSS and PASC, and their interaction with ACE2.

COVID-19 and sepsis survivors are at increased risk of cardiovascular events like stroke, heart attack, arrhythmia, and heart failure. In coronary heart disease, RAS promotes vascular inflammation and hypertension, leading to thrombosis and endothelial dysfunction. Cardioprotective roles are highlighted across several studies with ACE2 detected in cardiomyocytes, pericytes, and fibroblasts, thereby a consensus of cardioprotective effect of ACE2 (5, 27, 28). Genetic deletion of ACE2 was found to accelerate cardiac dysfunction by increasing local Ang II (29) which promotes vascular inflammation and hypertension associated with increased thrombosis, proliferation, and monocyte differentiation (30). Recruitment of inflammatory cytokines, proteases, coagulation factors, free radical species, and vasoactive intermediates, potentiate endothelial impairment, fibrous cap disruption, and thrombus formations. Animal models support the idea that Ang II induces endothelial dysfunction, while ACE inhibitors improve endothelial function in coronary heart disease (Figure 1) (31). Caution should be taken however, not to simplify the role of ACE2 in cardiovascular disorders.

### 3.3 Renal manifestations of PSS and PASC

Acute kidney injury (AKI) is a frequent and severe complication affecting around 50% of sepsis survivors, constituting 45–70% of all AKI cases, and 20–40% of COVID-19 survivors (32). Underlying causes stem from the systemic inflammatory response triggered by viral infections, disrupting kidney function, causing damage. Increased ROS production through RAS, and sympathetic nerve activation, lead to vasoconstriction and impaired renal blood flow. In sepsis, AKI involves immune dysregulation, systemic inflammation, hemodynamic changes, and renal microvascular endothelial cell dysfunction (33). In PASC, kidney function is significantly reduced, and acute tubular injury is common, possibly exacerbated by hemodynamic instability. ACE2 in renal proximal tube facilitates accumulation of SARS-CoV-2 into renal epithelial cells, inducing inflammation and cytotoxicity (11). SARS-CoV-2 binding to ACE2 is considered to cause the downregulation of ACE2 resulting in elevated Ang II and reduced Ang (1–7) (34), promoting inflammation, cytokine storms, and endothelial dysfunction, and renal damage. Viral entry and replication also increase hypercoagulation, leading to cardiac dysfunction, venous congestion, arterial underfilling, and AKD (35).

In sepsis survivors, decreased ACE2 expression in renal epithelial cells exacerbates tubular injury. Ang II, a potent mediator of renal inflammation, triggers renal injury through ROS elevation and inflammation via the NF-kB signalling pathway and chemokine expression. ROS-induced cell apoptosis, mediated by TLR4, has been observed. Ang II also upregulates TLR4 mRNA expression, potentially dependent on AT1R activation (23).

### 3.4 Neurological manifestations of PSS and PASC

Cognitive impairment is a common issue after critical illness or extended hospitalization. Both PSS and PASC patients, several months after the initial infection, experience fatigue, concentration problems, anxiety, slow processing, poor sleep, post-traumatic stress disorder, and depression (33, 36–38). Sepsis-induced cognitive decline arises from cerebral ischemia, neuronal dysfunction, and neuroinflammation, reduced cerebral blood flow, and neurotransmission impairment (37). Microglia and astroglia activation sustained neuroinflammation (39). In COVID-19, astrocyte reactivity during and after the acute phase leads to neuronal dysfunction, causing depression, anxiety, and memory problems. Mitochondrial dysfunction impact on neural tissue may be the primary contributor to neurological consequences. Hyperactivity of the RAS and neurogenic hypertension are also potential factors. Connections between brain RAS and memory and cognitive functions are emerging (40).

## 4 Potential of a unified therapeutic approach

Different therapeutic approaches have been tried and reported in the literature so far for both PSS and PSS. These are illustrated in Table 1.

**Table 1:** Selected studies on effector molecules acting on the primary systems and immunological pathways affected by sepsis and PSS.

Study	Objectives	Design	Key findings	Future opportunities
Yeh et al. (2022) (42)	Impact of enteral cholecalciferol and/or intravenous calcitriol on: CD4+ T cell subsets balance RAS - Severity of ALI	Obese mice models with polymicrobial sepsis. Four groups: Without Vitamin D With oral cholecalciferol With iv calcitriol With both cholecalciferol and iv calcitriol	Both forms of Vitamin D reduced lung injury IV calcitriol after sepsis: Better homeostasis of circulating T cells Upregulation RAS-associated anti-inflammatory gene expression Reduction of inflammatory mediator expression in the lungs Combined oral cholecalciferol before and IV calcitriol after sepsis: No synergistic effect on T-cell homeostasis No reduction of lung injury	Investigate of mechanisms beyond anti-inflammatory properties of vitamin D that provide protective effects against sepsis-induced lung injury
Abdel-Fattah et al. (2021) (52)	Protective effects of xanthenone on gentamicin-induced nephrotoxicity	Rats model, 4 groups: Normal control Xanthenone Gentamicin - Xanthenone + gentamicin	Xanthenone: Improves renal function Elevates antioxidant defense and limits oxidative stress in renal injury Attenuates inflammatory response Upregulates ACE2/Ag-(1–7) signaling with downregulation of Ag-II in renal injury Counteracted histopathological aberrations of kidney tissues	Efficacy of xanthenone as an adjunct therapy for managing gentamicin-associated renal damage
Ye et al. (2020) (45)	Protective effect of ACE2 on LPS-induced ALI	Murine models of ALI	Paired inhibition of LPS-TLR4 pathway and ACE2: Maintained dynamic balance of RAS Ameliorated LPS-induced ALI Overexpression of ACE2 inhibits inflammation and the LPS-TLR4 pathway	ACE2 overexpression comparable to ACE blockade
Li et al. (2015) (60)	Upregulation of ACE2 in preventing LPS–induced pulmonary inflammation and cytotoxicity	Murine models of ALI Generation of recombinant ACE2	ACE2 overexpression attenuated apoptosis and IL–1b and TNF–a secretion caused by LPS ACE2/Ang (1–7)/Mas axis inhibits the JNK/NF–kB pathways	Prevention of ALI by Ang (1–7)/Mas receptor through MAPK/NF–kB signalling pathways *in vivo*
Li et al. (2015) (19)	Efficacy of captopril on preventing LPS-induced ALI	Murine models of ALI	Protective effect of captopril in LPS-induced ALI: Regulates local RAS activity Suppresses inflammatory response Regulates balance of ACE and ACE2 expression	Investigate the mechanisms underlying the regulation of pulmonary ACE2 expression in LPS-induced ALI
Imai et al. (2005) (46)	Effect of ACE2 gene deficiency on sepsis-induced ALI using caecal ligation and perforation	Murine models of ALI Rescue experiment used recombinant human ACE2 protein (rhuACE2)	ALI results in decreased ACE2 expression and increased production of Ang II Depletion of the ACE2 gene aggravated lung injury Deficiency of ACE improved disease Treatment with rhuACE2 protein attenuated lung injury	Hydrostatic oedemas cannot be excluded, and the effects of local Ang II production on lung blood vessels require further investigation
Treml et al. (2010) (17)	Therapeutic potential of ACE2 in piglet model	Prospective, randomized, double-blinded animal study using piglets	ACE2 attenuates: Arterial hypoxemia Pulmonary hypertension Redistribution of pulmonary blood	Further studies in human models
Gomez-Mendoza et al. (2019) (27)	Molecular mechanisms involved in cardioprotective role of Ang II The impact of oral treatment with Ang-(1–7) on cardiac proteome dysregulation due to experimental myocardial infarction	Murin models of myocardial infarction	HPβCD/Ang-(1–7) combination treatment ameliorates the post-infarction condition due to the modulation of proteins that initially favour the resolution of inflammation and mitochondrial dysfunction Ang-(1–7) treatment after experimental myocardial infarction leads to the downregulation of the C-X-C chemokine receptor type 4 (CXCR4)	Further studies in human models
Yamamoto et al. (2006) (29)	Role of ACE2 in response to pressure overload	Murine models of pressure overload in ACE2/y mice and wild-type (WT) mice receiving transverse aortic constriction	Excessively increased cardiac Ang II plays a primary role in the activation of signal transduction in pressure overload– induced hypertrophy of ACE2/y mice	Further studies in human models
Chen et al. (2022) (32)	Role of STAT3 activation in early-stage AKI	Murine models of AKI	pSTAT3 expression was significantly positively related to ACE2 expression in AKI-mice Endogenous increase of ACE2 expression upregulated by STAT3 Activation in early-stage AKI plays protective role against acute tubular injury	Further studies in human models
Lankadeva et al. (2018) (54)	Role of Ang II on intrarenal pO2 in ovine sepsis	Animal model of sepsis	Ang II improved renal function without worsening medullary hypoxia	Further studies in human models
Tumlin et al. (2018) (55)	Role of Ang II on the outcomes of AKI needing RRT	Double-blind, randomized, placebo trial. *Post hoc* analysis of the angiotensin II for the treatment of high-output shock 3 trial in ICU setting Patients with AKI treated with renal replacement therapy at initiation of angiotensin II or placebo	RRT liberation greater in the Ang II group versus the placebo group	Patients with vasodilatory shock and AKI requiring RRT may benefit from Ang II

RAS, renin angiotensin system, ALI, acute lung injury; iv, intravenous; ICU, intensive care unit; LPS, lipopolysaccharide; AKI, acute kidney injury; Ang II, angiotensin II; RRT, renal replacement therapy.

However, there is a need for a comprehensive approach to treating PSS and PASC, considering both immunomodulation and specific health issues. RAS plays a significant role in immunomodulation through various pathways.

Some studies target T-cell immunity directly. IL-7, a cytokine that promotes T-cell development and activation, has shown promise in increasing T-cell expression and function in sepsis since it increases CD4+ and CD8+ T-cell expression in murine models and improve lymphocyte function (41). Additionally, reduced capacity of T-cells producing the antimicrobial IFN-γ led to consideration of IFNs as immunostimulatory agents. However, excessive IFN-γ has been identified as a key factor in immunosuppression, leading to questions about its therapeutic use (20). Finally, calcitriol affects Treg homeostasis, balancing CD4+ T cell subsets and reducing viral-induced inflammation (42).

While sepsis patients exhibit high cytokine levels, no established biomarker panel exists for PSS. As a result, the focus has shifted towards immune modulation, given the shared manifestations and immunological mechanisms between PASC and PSS. Finding a suitable therapeutic option for one condition may open pathways for the treatment of the other. Specifically, dysregulation of inflammation by RAS is well studied in sepsis (6) and COVID-19 (43), and research is shedding light on its role in PSS and PASC. Accordingly, intervening in the RAS pathway to regulate the dysfunctional inflammatory response associated with PSS and PASC holds significant potential.

## 5 Regulation of RAS as a unified therapeutic approach

ACE2 is distributed throughout the endothelium where it is involved in several pathophysiological events. When bound with SARS-CoV-2 with ACE2 the normal conversion of Ang II to Ang (1–7) becomes inhibited, increasing Ang II activity. Addition of a synthetic Ang (1–7), TXA-127, achieves RAS balance (44). Other RAS inhibitors need to be further investigated as a great opportunity to have effective treatment in PASC.

### 5.1 RAS in pulmonary manifestations

Other trials demonstrated ACE inhibitors (ACEIs) and ACE receptor blockers (ARBs) restore normal lung function in mice, indicating utility for COVID-19-induced ARDS (19, 45, 46). However, administration of ACE2Is/ARBs could enhance entry of SARS-CoV-2 by increasing ACE2 expression. To avoid this, Golab et al. (47) describe potential for using a recombinant human ACE2 (rhACE2) to compete with SARS-CoV-2, reduce viral entry and replication thereby alleviating lung infection-induced damage. Interestingly, IFNs have shown antiviral properties, limiting replication of SARS-CoV-2 in bronchial epithelial cells, implying their usefulness to counteract increased ACE2. Despite IFN inducing ACE2 expression, it was not enough to enhance viral replication of SARS-CoV-2 in presence of IFN antiviral activity (48). While extrapolation of these findings to other biological tissues is not possible, *in vivo* experiments, along with recent studies, support inducing expression of ACE2 for short-term antiviral therapies (49–51).

Intravenous administration of ACE2 has shown to effectively mitigate the LPS-induced overexpression of pro-inflammatory cytokines, including IL-1b, IL-6, and TFN-a (45). Studies involving mice infected with H5N1 influenza virus, the use of rhACE2 not only reduced replication in the lungs but also led to a decrease in mortality rates (26). Similarly, when Ang (1–7) was administrated to rats exposed to acid aspiration and high stretch ventilation reduced the number of inflammatory cells in bronchoalveolar lavage and attenuated pulmonary fibrosis 2 weeks after the acid aspiration injury (35). The beneficial effects of Ang 1–7 administration in LPS-induced lung injury were related to a downregulation of AT1R (30). These approaches vary among different phenotypes, with corticosteroids and immune modulators showing promise for COVID-19-related ARDS.

### 5.2 RAS in cardiovascular and renal manifestations

Increased ACE2 alleviates acute tubular injury in mice (32, 52, 53). Ang II increases intraglomerular pressure, filtration, and creatinine clearance, maintaining renal function during infection. Studies showed infusion of Ang II improved renal function without deteriorating intrarenal oxygenation (54) without association with increased risk of AKI and reduced vasodilatory shock (55, 56). These highlight insights into using Ang II to restore renal function following viral infection.

ACE counterbalances ACE2, limiting the potent vasoconstrictive effect of Ang II. Evidence from animal and clinical trials show apparent beneficial effects of simultaneous activation of ACE2/Ang (1–7)/MasR and inhibition of ACE/AngII/AT1R arms, could provide a valid approach for tackling organ dysregulation associated with PSS and PASC through modulation of the pro- and anti-inflammatory responses. Heterogeneity of PSS and PASC and their complex interaction with different RAS elements provoke exploration of combination therapy for full restoration of RAS functioning using administration of rhACE2, Ang (1–7), and Ang (1–9), with careful regulation of genetic and protein ACE2 expression using selected IFNs. Moreover, supported by the beneficial effects of ARBs and ACEIs in PTSD (57).

Numerous drugs caused adverse effects (58), as such existing techniques for treating CHD have considerable disadvantages. As a result, novel, safe, and effective therapeutic pharmacotherapies targeting the RAS and the discovery of molecular biomarkers to detect the course of CHD in its early stages are required. If CHD is diagnosed, its progression should be followed to prevent and/or treat secondary HF. Here, the prevention usually contains medication effective in the RAS, e.g., ACEi, ARB, or aldosterone antagonists, by which the development of CHF can be delayed (59).

The impactful nature of the immunomodulator RAS arm ACE2/Ang (1–7)/MasR in sepsis begun to reveal its true protective potential due to the arrival of COVID-19. Based on our research, there is great promise in developing the research into non-inflammatory cellular and molecular mechanisms of ACE2 expression and/or inhibition for PSS and PASC due to its ubiquitous presence and profound involvement in developing certain manifestations of these post-acute syndromes.

## 6 Conclusion

Treatment for PSS and PASC focuses on rehabilitation and symptom management, with potential shared therapeutic approaches. However, the absence of clear consensus definitions for both conditions, along with limited knowledge about specific effector molecules, hinders unified treatment. To improve outcomes, a comprehensive, tailored therapy begins with symptom recognition, risk factor identification, and understanding underlying causes. Standardization is crucial, and more research funding and expertise can support clinical trials. Challenges in investigating post-infection conditions include high loss to follow-up and a lack of validated measurement standards. Complexity of the immune response makes finding a comprehensive treatment approach challenging, as current strategies primarily target individual conditions.

## Author’s note

Views and opinions expressed are however those of the author(s) only and do not necessarily reflect those of the European Union or the END VOC Consortia. Neither the European Union nor the granting authority can be held responsible for them.

## Author contributions

DH: Investigation, Writing – original draft. MC: Conceptualization, Supervision, Writing – review & editing. EP: Supervision, Writing – review & editing. AG: Conceptualization, Supervision, Writing – review & editing.
